# Mechanism of histone demethylase KDM5A in osteoporotic fracture healing through epigenetic regulation of the miR-495/SKP2/Runx2 axis

**DOI:** 10.1186/s10020-025-01098-5

**Published:** 2025-02-19

**Authors:** Zhuoran Li, Junyan Zhang, Tingting Xu, Zhiying Hao, Yadong Li

**Affiliations:** 1 School of Medicine, University of Nottingham, Nottingham, NG7 2NR UK; 2 Department of Affiliated Cancer Hospital, Shanxi Medical University, Taiyuan, 030001 China; 3 Department of Pharmacy, Shanxi Medical University, Taiyuan, 030001 China; 4 Department of Pharmacy, Shanxi Hospital Affiliated to Cancer Hospital, Shanxi Province Cancer Hospital, Chinese Academy of Medical Sciences/Cancer Hospital Affiliated to Shanxi Medical University, No. 3, ZhiGong New Street, Xinghualing District, Taiyuan, Shanxi Province 030013 China; 5 Department of Emergency, The Second Hospital of Shanxi Medical University, No. 382 Wuyi Road, Xinghualing District, Taiyuan, Shanxi Province 030001 China

**Keywords:** Fracture healing, Histone demethylase, KDM5A, Epigenetics, miR-495, SKP2, Runx2, Osteoporosis

## Abstract

**Background:**

Osteoporosis represents a salient metabolic bone disorder. Histone demethylase plays a vital role in bone development and homeostasis. This study explored the mechanism of histone demethylase KDM5A affecting osteoporotic fracture healing via the miR-495/SKP2/Runx2 axis.

**Methods:**

The murine model of osteoporotic fracture was established. The bone mineral density, maximum elastic stress, and maximum load were tested. The relative trabecular bone volume, bone trabecular thickness, and trabecular number at the proximal end of tibia were detected. The histopathological changes of femur tissues and bone microstructure were observed. Expressions of KDM5A and osteogenic factors were detected. The cell proliferation, alkaline phosphatase activity, and calcified nodules were measured. The binding relationships between KDM5A and miR-495 promoter, and miR-495 and SKP2 were verified. The interaction between SKP2 and Runx2 was detected. The ubiquitination level of Runx2 and the stability of Runx2 protein were detected.

**Results:**

KDM5A was highly expressed in the murine model of osteoporotic fracture. Interference of KDM5A expression facilitated fracture healing in osteoporotic mice. KDM5A downregulated miR-495 expression by promoting the H3K4me3 methylation of the miR-495 promoter. Inhibition of miR-495 reversed the effect of KDM5A silencing on osteoblast proliferation, differentiation, and mineralization. miR-495 facilitated osteoblast proliferation, differentiation, and mineralization by targeting SKP2. SKP2 suppressed Runx2 expression through ubiquitination degradation. Inhibition of Runx2 reversed the promoting effect of SKP2 silencing on osteogenic differentiation.

**Conclusion:**

KDM5A attenuated the inhibition of miR-495 on SKP2 and promoted the ubiquitination degradation of Runx2 protein by SKP2, thereby repressing osteoblast differentiation and retarding osteoporotic fracture healing.

## Background

Osteoporosis represents a systemic skeletal disorder that results in bone fragility and fracture susceptibility (Kerschan-Schindl 2016). Multiple risk factors such as nutrition, inflammation, mechanical stress, and hormone fluctuations are implicated in the occurrence or development of osteoporosis (Wang et al. 2020b). Clinically, osteoporosis is mainly presented with somatic pain, hunchback and brittle fractures, especially the proximal femoral fractures in the elderly (Shen et al. 2019). Osteoporotic fracture is a common complication of osteoporosis, which is caused by bone mass reduction and bone microstructure deterioration (Wang et al. 2016a). Osteoporotic fracture healing is impaired as the global aging of the population, which seriously threatens public health (Varanasi et al. 2011). The promotion of osteoporotic fracture healing is of great significance to shorten the length of hospital stay and reduce the related complications (Cheung et al. 2021). Therefore, exploring reasonable and potent approaches to facilitate osteoporotic fracture healing remains an urgent issue.

Epigenetic mechanisms, such as DNA methylation, histone modification, and noncoding RNAs, can modulate gene expression without altering genetic sequence, and aberrant epigenetic patterns lead to pathological conditions, including cartilage and bone diseases, and age-related diseases (Huang et al. 2018). Epigenetic factors play a vital role in the pathogenesis of skeletal disorders, including osteoporosis (van Meurs et al. 2019). Histone methylation on arginine or lysine is abundant on the N- and C- terminal tails of histones, which can turn on or off transcription according to the degree and position of methylation (Jose et al. 2020). Histone methylation can be eliminated by histone demethylases (KDMs) (Wang et al. 2016b). The KDM5 subfamily is composed of four histone demethylases (KDM5A, KDM5B, KDM5C, and KDM5D), participating in a variety of human biological processes, such as genomic stability, hormone response, stem cell regeneration, as well as cell proliferation and differentiation (Shi and Whetstine 2007). Microarray analysis shows that KDM5A is upregulated in osteoporosis, which downregulates Runx2 by modifying its promoter H3K4 methylation and consequently causes impaired bone formation (Kirtana et al. 2020). KDM5A inhibits osteogenic differentiation by the regulation of H3K4me3 and H3K27ac of SOCS1 (Zhang et al. 2020). Knockdown of KDM5A restores osteoporotic bone mesenchymal stem cell (BMSC) lineage commitment by enhancing H3K4me3 level, and KDM5A-mediated H3K4me3 modification is implicated in the pathogenesis of osteoporosis (Wang et al. 2016b). However, the specific mechanism of KDM5A in osteoporotic fracture healing remains further exploration.

microRNAs (miRNAs) are small noncoding RNAs (about 18–25 nucleotides in length) that can be modulated by epigenetic modification (Yao et al. 2019). miRNAs act as diagnostic biomarkers and represent the targets for the treatment of bone loss and fracture healing in osteoporosis patients (Seeliger et al. 2014). KDM5A has been demonstrated to bind to the miR-495 promoter (Du et al. 2020). miR-495 represses inflammatory response and enhances bone differentiation of human fibroblast-like synovial cells, thereby delaying ankylosing spondylitis progression (Du et al. 2019). Accordingly, we speculated that KDM5A can affect osteoporotic fracture healing through epigenetic regulation of miR-495. We herein investigated the underlying role and mechanism of KDM5A in osteoporotic fracture healing, which shall confer novel insights for the promotion of osteoporotic fracture healing from an epigenetic aspect.

## Materials and methods

### Ethics statement

This study got the approval of the Ethical Committee of Shanxi Province Cancer Hospital. All the animal experiments were implemented on the guide for the care and use of laboratory animals and on minimized animal number and the least pains.

### The murine model of osteoporotic fracture

Totally 32 female C57BL/6J mice (aged 6–8 weeks, weighing 22–27 g) were purchased from SJA Laboratory Animal Co., Ltd (Changsha, Hunan, China). The mice were reared in separate cages at 22–24℃ and maintained in a 12 h light/dark cycle. Food and water were provided ad libitum. Mice were randomly assigned to 4 groups based on the body weight using the random number table: Sham group, Model group, sh-NC group, and sh-KDM5A group, with 8 mice in each group. Except for the sham-operated mice, the other mice underwent ovariectomy to establish the osteoporosis model of estrogen deficiency (Lu et al. 2021).

The osteoporotic mice were used to establish the femoral fracture model. A C-shaped instrument applying three-point bending was used to generate transverse femoral shaft fracture. The mouse right knee was exposed by lateral parapatellar approach, and the patella medially was dislocated. The femoral intercondylar groove was exposed by full flexion at the knee joint, and a burr hole with a diameter of 0.5 mm was made in the center of the intercondylar groove. A 24-gauge needle with a diameter of 0.5 mm was inserted into the burr hole to avoid obvious displacement of the fracture and keep the fracture site neat and stable, and the needle tip passed through the top of the greater trochanter. After minimal lateral exposure, a thin saw with a depth of 3 mm was applied to the intermediate shaft to weaken the bone and prevent complex fractures. The right femur of each mouse was fractured by three-point bending, and then the myofascial and skin were closed (Furuta et al. 2016). During the surgery, all animals were kept warm on a heating pad. After the surgery, Buprenorphine (0.1 mg/kg; Buprenovet^®^, Elanco, Greenfield, IN, USA) was administered for pain relief.

KDM5A lentivirus interference vector and its negative control (NC) were injected into mice via tail vein (Shimizu et al. 2012), with the titer of 1.0 × 10 TU/mL and the injection volume of 3 µL 9 (Wang et al. 2020a). The mice were sacrificed after 3% pentobarbital sodium anesthesia. The bilateral femur was separated from the hip and knee joint by surgical shear, and the muscles and fascia were removed. The bone mineral density (BMD), the maximum elastic stress, and the maximum load were tested. Part of the femur tissues was stored at -20 ℃ for molecular determination; the rest of the femur tissues was fixed with 4% neutral formaldehyde at 4℃ overnight, decalcified with EDTA for 2 weeks, embedded in paraffin, and sectioned for staining.

### Detection of BMD

BMD of the right femur of mice in each group was measured by a dual-energy X-ray absorptiometry (Ultrafocus 100, Faxitron, USA). For the detection of maximum elastic stress and maximum load, the spacing between the two columns of the material testing machine was adjusted to 20 mm. The right femur samples of mice were placed between two columns, and the two columns supported the two ends of femur. The direction and position of femur samples were consistent to reduce the system measurement error. The upper-pressure head of the material testing machine was close to the femur and descended at a speed of 1 mm/min. The three-point bending test was carried out until the fracture of femur occurred. The experimental data were recorded at the sampling frequency of 10 times per second through the SMD digital signal acquisition and testing system. The data were automatically saved in the software, and the software recorded the maximum elastic stress and maximum load (Dempster et al. 2013).

### Analysis of micro computed tomography (Micro-CT)

The right femurs of the designated mice were removed, fixed in 10% neutral formalin buffer, and scanned by Micro CT-35 (Scanco Medical, Bassersdorf, Zurich, Switzerland) with the space resolution of 7 μm. The three-dimensional reconstruction image was obtained from the contouring two-dimensional image by the distance transformation method based on binary image. The region of interest (ROI) of femur (constant height was 3.75 mm and width was 15.00 mm) was checked layer by layer on the tomographic image, from the proximal femoral head, femoral neck, and femur body to the lateral condyle and inner condyle at the distal end. Scanco software was used to quantify the parameters of trabecular bone, including relative trabecular bone volume (BV/TV), bone trabecular thickness (Tb.Th), and trabecular number (Tb.N) (Tang et al. 2017; Li et al. 2019b).

### Fluorochrome histomorphometry

CalceinHisto software (Liverpool, UK) was used for histological analysis. Three histological measurements were calculated: mineral apposition rate (MAR), ratio of mineralized surface to bone surface (MS/BS), and bone formation rate (BFR/BS). MAR (µm/day) is the distance between two labels divided by the time between label administration. MS/BS (%) is the ratio of label surface to bone surface. BFR/BS (µm^3^ /µm^2^ /day) is the product of MS/BS and MAR.

### Hematoxylin and eosin (H&E) staining

The femur tissue samples in each group were washed with normal saline, fixed in 4% paraformaldehyde for 30–50 min, and then washed, dehydrated, cleared, waxed, embedded, and sectioned. The tissue sections were put on the glass slides, dried at 45℃, dewaxed, then treated with gradient alcohol, and washed with distilled water for 5 min. Afterward, the sections were stained with hematoxylin for 5 min, rinsed with running water for 3 s, differentiated with 1% hydrochloric acid ethanol for 3 s, stained with 5% eosin solution for 3 min, and then dehydrated, cleared and sealed. The sections were observed under a microscope.

### Safranin O/fast green staining

The paraffin sections were placed in 60℃ oven overnight, treated with xylene I and II solution for 5 min respectively, 100% alcohol for 2 min, 95% and 80% alcohol and deionized water for 1 min respectively. The A and B solution mixture was added, and the floating color was washed off after 3 min. The sections were differentiated in hydrochloric acid ethanol for 1 min, washed with running water for 3 min, then stained with fast green solution, and differentiated in acetic acid solution for 30 s. The sections were stained with 1% safranin O for 6 min, then washed with 95% alcohol, dehydrated, cleaned, and sealed. Five sagittal discontinuous sections were randomly selected from each femur sample, and the images were collected by the micro digital camera (DP80, Olympus Optical Co., Ltd, Tokyo, Japan).

### MC3T3-E1 cell culture and treatment

Mouse osteoblast cell line MC3T3-E1 was provided by Sigma-Aldrich (Merck KGaA, Darmstadt, Germany) and incubated in α-modified Eagle’s medium (α-MEM) (Thermo Fisher Scientific, Waltham, MA, USA) containing 10% fetal bovine serum (FBS) (PAA Laboratory, Germany), 1% penicillin/streptomycin (Gibco, Grand Island, NY, USA), and 1% L-glutamine (Biochrom, Merck, Berlin, Germany). The medium was supplemented with 10 mM β-glycerophosphate and 0.2 mM ascorbic acid-2-phosphate (both from Sigma-Aldrich) to induce osteogenic differentiation (Xiong et al. 2015; Furuta et al. 2016). Lipopolysaccharide (LPS)-induced MC3T3-E1 cells were infected with corresponding lentivirus (sh-NC, sh-KDM5A, sh-SKP2, sh-Runx2, oe-SKP2, oe-NC) (GeneChem, Shanghai, China), and the multiplication of infection was 20 (Zhang et al. 2017). The subsequent experiments were conducted after 48 h. miR-495 inhibitor, miR-495 mimic, and corresponding negative controls (inhibitor NC, mimic NC) were provided by GeneChem and transfected into MC3T3-E1 cells according to the manufacturer’s instructions of Lipofectamine 3000 (Invitrogen), and subsequent experiments were conducted 48 h after transfection.

### Cell counting kit-8 (CCK-8) assay

The cells were seeded into 96-well plates (1 × 10 cells/well), and cultured in 100 µL medium 3 containing 10% FBS for 1–5 days. The number of cells was determined by CCK-8 assay kit (Dojindo Molecular Technologies, Gaithersburg, Maryland, USA). Then, 10 µL CCK-8 solution was added to each well for 1 h-incubation, and the absorbance at 490 nm was measured by a microplate reader.

### Alkaline phosphatase (ALP) staining

MC3T3-E1 cells were seeded into 12-well plates (2 × 10 cells/well). After 7 days of 5 incubation in osteogenic medium, the cells were collected and stained with BCIP/NBT ALP chromogenic reagent (CWBIO, Beijing, China). The staining dish was washed with phosphate-buffered saline (PBS) 3 times and then observed and photographed under the microscope. To determine ALP activity, the cells were rinsed with PBS three times, lysed with 1% Trix-100 (Sigma-Aldrich) on ice for 10 min, and then centrifuged at 12,000 rpm and 4 ℃ for 30 min. The protein concentration was measured using bicinchoninic acid (BCA) assay kit (Prod#23225, Pierce Thermo Scientific, Waltham, MA, USA), and ALP activity was evaluated using ALP assay kit (A059-2, Nanjing Jiancheng Bioengineering Institute, Nanjing, China).

### Alizarin red staining

After 21 days of incubation, the cells were subjected to alizarin red staining using bone mineralization detection kit (Sigma-Aldrich). The cells were stained with 2% alizarin red at pH 4.2 for 10 min and then washed with distilled water. The mineralized nodules were examined by a phase contrast microscope. For mineralization quantification, the cells were treated with ultrasound in PBS/Triton/HCl and then incubated overnight in 5 µL HCl 6 M at 4℃. After centrifugation at 1500 × g for 5 min, the supernatant was transferred to 96-well plates, and the protein content was treated with calcium test solution. Ammonium hydroxide was added to each well, and the absorbance at 595 nm was measured by a microplate reader (Lu et al. 2021).

### Dual-luciferase assay

The predicted binding site fragments and mutation fragments of miR-495 promoter and KDM5A were inserted into luciferase reporter vector as reporter plasmids miR-495 promoter-WT (3’-CAGTGACCCA-5’) and miR-495 promoter MUT (3’-TGAGAGTATG-5’). oe-NC or oe-KDM5A was transfected with luciferase reporter plasmids of miR-495 promoter into 293T cells (Oulu Biotecnology, China) respectively to detect whether KDM5A could bind to the miR-495 promoter. After 48 h-transfection, the cells were collected and lysed, and detected by luciferase detection kit (K801-200, BioVision Inc., Mountain View, CA, USA). The luciferase reporter gene was detected by dual-luciferase reporter gene analysis system (Promega, Madison, Wisconsin, USA). Ranilla luciferase was used as an internal reference gene. According to the ratio of firefly luciferase measured relative luciferase activity (RLU) divided by ranilla luciferase measured RLU, the activation degree of the target reporter gene was compared. The predicted SKP2 mRNA 3’UTR and the binding site fragments and mutation fragments of miR-495 were inserted into the luciferase reporter vectors as reporter plasmids SKP2-WT and SKP2-MUT. mimic NC and miR-495 mimic were co-transfected with SKP2 mRNA luciferase reporter plasmids to detect whether miR-495 could bind to SKP2 mRNA 3’UTR. The specific steps were the same as above.

### Chromatin immunoprecipitation (ChIP) assay

When reaching 70–80% confluence, the cells were fixed with 1% formaldehyde for 10 min to make the DNA and protein cross linked, and then the cells were randomly broken by ultrasonic treatment for 15 times, with 10 s each time and 10 s interval. After centrifugation at 10,000 × g and 4℃, the supernatant was collected and divided into two tubes. NC antibody rabbit anti-IgG (ab109489, 1:100, Abcam Inc., Cambridge, MA, USA), rabbit KDM5A antibody (use 2 µg for 25 µg of chromatin, Abcam), and target protein specific antibody rabbit anti-H3K4me3 (ab32356, use 2 µg for 25 µg of chromatin, Abcam) were added for incubation at 4℃ overnight. Endogenous DNA-protein complexes were precipitated by protein agarose/sepharose. After a short centrifugation, the supernatant was discarded. The nonspecific complexes were washed and de-crosslinked overnight at 65℃. DNA fragments were extracted and purified with phenol/chloroform. Quantitative real-time polymerase chain reaction (qRT-PCR) was used to detect the enrichment of KDM5A in miR-495 promoter and H3K4me3 in KDM5A promoter.

### Immunoprecipitation (IP)

Briefly, 10^7^-10^8^ cells were detached with trypsin and collected, added with 1 mL radio-immunoprecipitation assay (RIPA) buffer, and lysed on ice for 30 min. The supernatant was collected after centrifugation at 4℃ and 7000 g for 20 min. Then, 100 µL supernatant was taken as input, and the remaining supernatant was incubated with 30 µL protein A + G agarose at 4 ℃ for 1 h for pre-clearance. After centrifugation at 4℃ and 700 g, the same volume of supernatant was transferred to two EP tubes. One tube was added with 1 µg Runx2 protein antibody, and the other tube was added with 1 µg IgG, followed by incubation overnight at 4℃. Next, 50 µL protein A + G agarose was added to each tube. After incubation at 4 ℃ for 1–2 h, the precipitates were collected by centrifugation and washed with IP wave buffer 3 times. The corresponding amount of 2 × SDS sample buffer was added to the precipitation and input, and the samples were boiled and loaded for Western blotting (WB) (Gassen et al. 2019).

### Ubiquitin pull-down assay

MC3T3-E1 cells were lysed in 500 µL IP buffer at 4℃ for 30 min. After centrifugation, the supernatant was cultured overnight with FlagM2 agarose beads at 4℃. His ubiquitin and Flag-labeled Runx2 and Flag-labeled SKP2 were transfected into cells. After 48 h, the cells were harvested and the amount of Flag-Runx2 in the lysate was balanced. The lysate was incubated overnight with Ni-NTA beads (Qiagen GmbH, Hilden, Germany) at 4℃. The magnetic beads were washed and the IP mixture was boiled in 2 × SDS loaded buffer in the presence of 200 mM imidazole. The eluted proteins were analyzed using Western blot and Flag-HRP (Sigma-Aldrich) probe was used for the detection of Runx2 ubiquitination (Zhang et al. 2014).

### Cycloheximide chase assay

For the detection of Runx2 protein stability, SKP2 was overexpressed in MC3T3-E1 cells and then the cells were incubated with 20 µg/mL CHX (protein synthesis inhibitor, Sigma-Aldrich). The cells were treated with RIPA lysate (P0013B, Beyotime, Shanghai, China) and centrifuged at 12,000 g/min for protein extraction. Then, the cells were lysed in RIPA buffer containing 0.1% SDS, followed by Western blotting. Runx2 level was quantified by Image J and normalized to Actin. The protein was extracted at 0 min, 30 min, 60 min, and 90 min, and Runx2 level was detected using WB. The experiment was repeated three times (Lee et al. 2017).

### Quantitative real-time polymerase chain reaction (qRT-qPCR)

Total RNA was extracted from cells and tissues using TRIzol reagent (15596026, Invitrogen, Carlsbad, CA, USA). The extracted RNA was reverse transcribed into cDNA using miRNA First Strand cDNA Synthesis (Tailing Reaction) kit (B532453-0020, Sangon Biotech, Shanghai, China). The synthesized cDNA was subjected to qRT-PCR using Fast SYBR Green PCR kit (Applied Biosystems, Carlsbad, CA, USA) and ABI PRISM 7300 qRT-PCR system (Applied Biosystems). Three duplicates were set for each well. The relative expression of gene was calculated by 2^−ΔΔCt^ method, with glyceraldehyde-3-phosphate dehydrogenase (GAPDH) as internal reference. The primers are shown in Table 1.

**Table 1:** Primer sequences

Gene name	Primer sequence
miR-495	F: 5′-GGGGAAACAAACATGGTGCAC-3′
	R: 5′-CAGTGCGTGTCGTGGAGT-3′
KDM5A	F: 5′-TTACCAACAGGTCAGACGCAT-3′
	R: 5′-GGTTTGCTACATTCCTCGGCG-3′
SKP2	F: 5’-AGTCTCTATGGCAGACCTTAGACC-3’
	R: 5’-TTTCTGGAGATTCTTTCTGTAGCC-3’
Runx2	F: 5’-GACTGTGGTTACCGTCATGGC-3’
	R: 5’- ACTTGGTTTTTCATAACAGCGGA − 3’
Osterix	F: 5’- AGGAGGCACAAAGAAGCCATAC − 3’
	R: 5’-AGGGAAGGGTGGGTAGTCATT-3’
OPN	F: 5’-GGAGTTGAATGGTGCATACAAGG-3’
	R: 5’-CCACGGCTGTCCCAATCAG-3’
OCN	F: 5’- CCCTGAGTCTGACAAAGCCT-3’
	R: 5’-GCGGTCTTCAAGCCATACTG-3’
U6	F: 5’- CGCTTC GGCAGCACATATAC − 3’
	R: 5’-TTCACGAATTTGCGTGTCAT-3’
GAPDH	F: 5’- ATCCACGGGAGAGCGACAT − 3’
	R: 5’-CAGCTGCTTGTAAAGTGGAC − 3’

Note: miR-495: microRNA-495; KDM5A: lysine-specific demethylase 5 A; SKP2: S-phase kinase-associated protein 2; Runx2: Runt-related transcription factor 2; OPN: osteopontin; OCN: osteocalcin; GAPDH: glyceraldehyde-3-phosphate dehydrogenase

### Western blotting

The tissues or cells of each group were added with lysate containing phenylmethylsulfonyl fluoride (PMSF), lysed on ice for 30 min, and centrifuged at 4℃ and 10,000 rpm for 15 min. The supernatant was collected and transferred to a new Eppendorf. BCA kit (Thermo Fisher Scientific) was used to determine the protein concentration. The total protein (30 µg) was subjected to polyacrylamide gel electrophoresis (constant voltage 80 V for 35 min and 120 V for 45 min). After electrophoresis, the protein was transferred onto the polyvinylidene fluoride membranes (Amersham, Arlington Heights, IL, USA), blocked with 5% skim milk for 1 h, and incubated with rabbit anti-KDM5A (ab249990, 1:1000, Abcam), H3K4me3 (ab213224, 1:1000, Abcam), SKP2 (ab183039, 1:200, Abcam), Runx2 (ab236639, 1:1000, Abcam), and GAPDH (ab181602, 1:10000, Abcam) overnight. Following PBST (PBS buffer containing 0.1% Tween-20) washing three times (10 min/time), the membranes were cultured with horseradish peroxidase-labeled goat anti-rabbit IgG (ab6721, 1:2000, Abcam) for 1 h. Image Pro Plus 6.0 (Media Cybernetics, Bethesda, MA, USA) software was used to scan the protein bands and analyze the relative expression of protein.

### Statistical analysis

Data analysis was introduced using the SPSS 21.0 (IBM Corp., Armonk, NY, USA) and GraphPad Prism 6.0 (GraphPad Software Inc., San Diego, CA, USA). Kolmogorov-Smirnov method checked that the data were in normal distribution. Measurement data are expressed as mean ± standard deviation. Unpaired t test was used for the comparisons between two groups. One-way analysis of variance (ANOVA) was employed for the comparisons among multiple groups, followed by Tukey’s multiple comparisons test. The *p* < 0.05 meant a statistical difference.

## Results

### KDM5A was highly expressed in the murine model of osteoporotic fracture

**Fig. 1:**
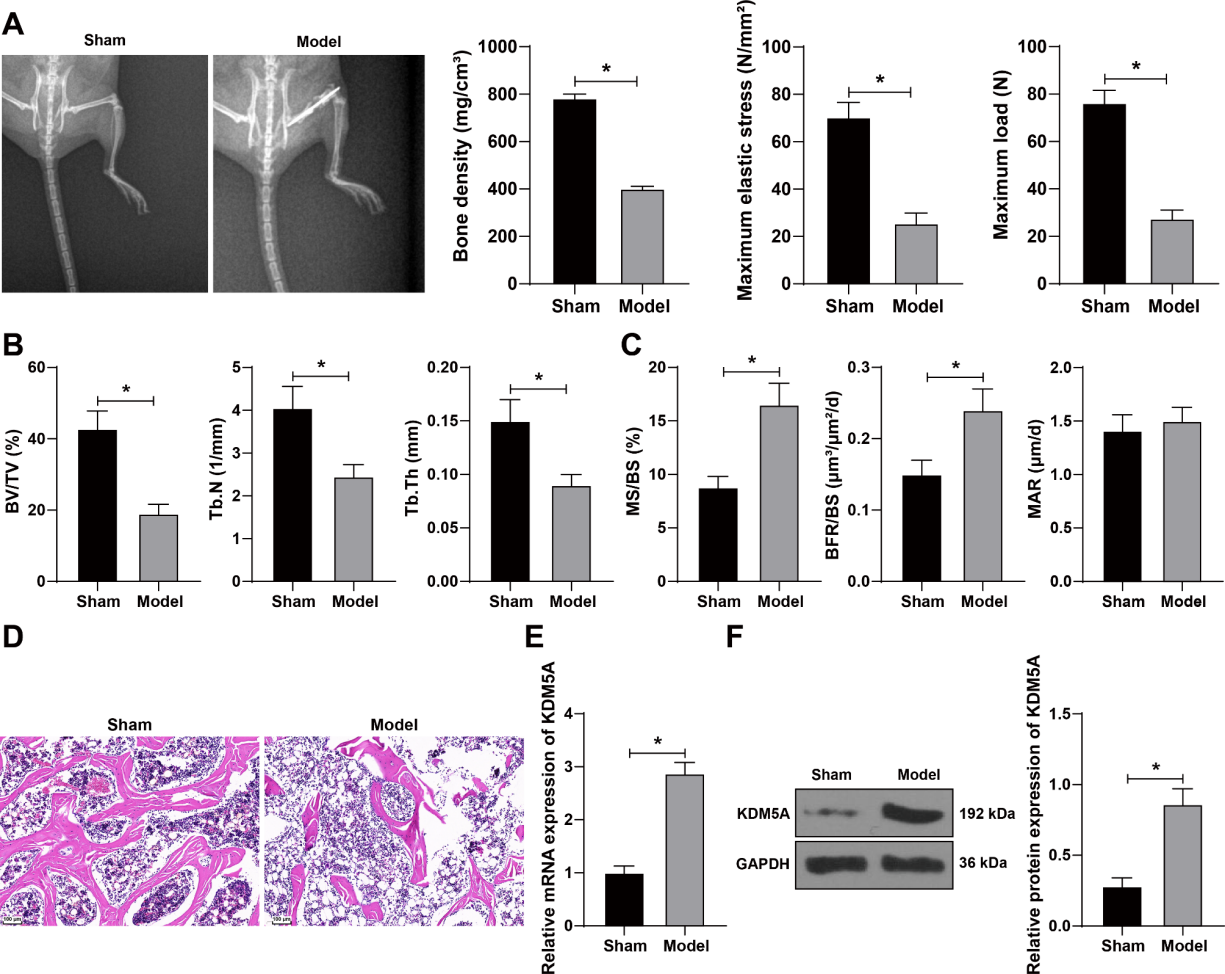
KDM5A was highly expressed in the murine model of osteoporotic fracture. **A**: The fracture was observed, and the bone mineral density, maximum elastic stress and maximum load were measured by dual-energy X-ray absorptiometry. **B**: The relative trabecular bone volume (BV/TV), bone trabecular thickness (Tb.Th), and trabecular number (Tb.N) were measured by Micro-CT. **C**: The dynamic osteogenic parameters in histomorphometry were examined. **D**: The histopathological changes of femoral tissues after modeling were observed by H&E staining. **E**: KDM5A expression in mouse femoral tissues was detected by qRT-PCR. **F**: KDM5A expression in mouse femoral tissues was detected by Western blotting. *P < 0.05. The measurement data were expressed by Mean ± SD, and the unpaired t test was used for comparisons between two groups. *N* = 8

To explore the effect of KDM5A on osteoporotic fracture healing, we established a murine model of osteoporotic fracture, observed the fracture by X-ray, and then measured the BMD, maximum elastic stress, and maximum load. Compared with sham-operated mice, the model mice showed clearly visible fracture line, and significantly reduced BMD, maximum elastic stress, and maximum load (all *p* < 0.05) (Fig. 1A). The BV/TV, Tb.Th, and Tb.N at the proximal end of tibia were measured by Micro-CT, and the results demonstrated that compared with sham-operated mice, the model mice showed reduced BV/TV and Tb.N, and thinned Tb.Th (all *p* < 0.05) (Fig. 1B). The dynamic osteogenic parameters in histomorphometry were detected. Compared with the sham-operated mice, the model mice showed a significant increase in MS/BS and BFR/BS (*p < 0.05*), but there was no statistical difference in MAR (Fig. 1C). HE staining showed that the trabecular bone of the model mice became thinner, in the presence of fracture; the space between trabecular bone became wider with irregular arrangement (Fig. 1D). These results demonstrated that the murine model of osteoporotic fracture was successfully established. Then, KDM5A expression in mouse femur was detected using qRT-PCR and WB. The results exhibited that KDM5A was upregulated in the femur tissues of osteoporotic fracture mice (all *p* < 0.05) (Fig. 1E-F).

### Interference of KDM5A facilitated fracture healing in osteoporotic mice

**Fig. 2:**
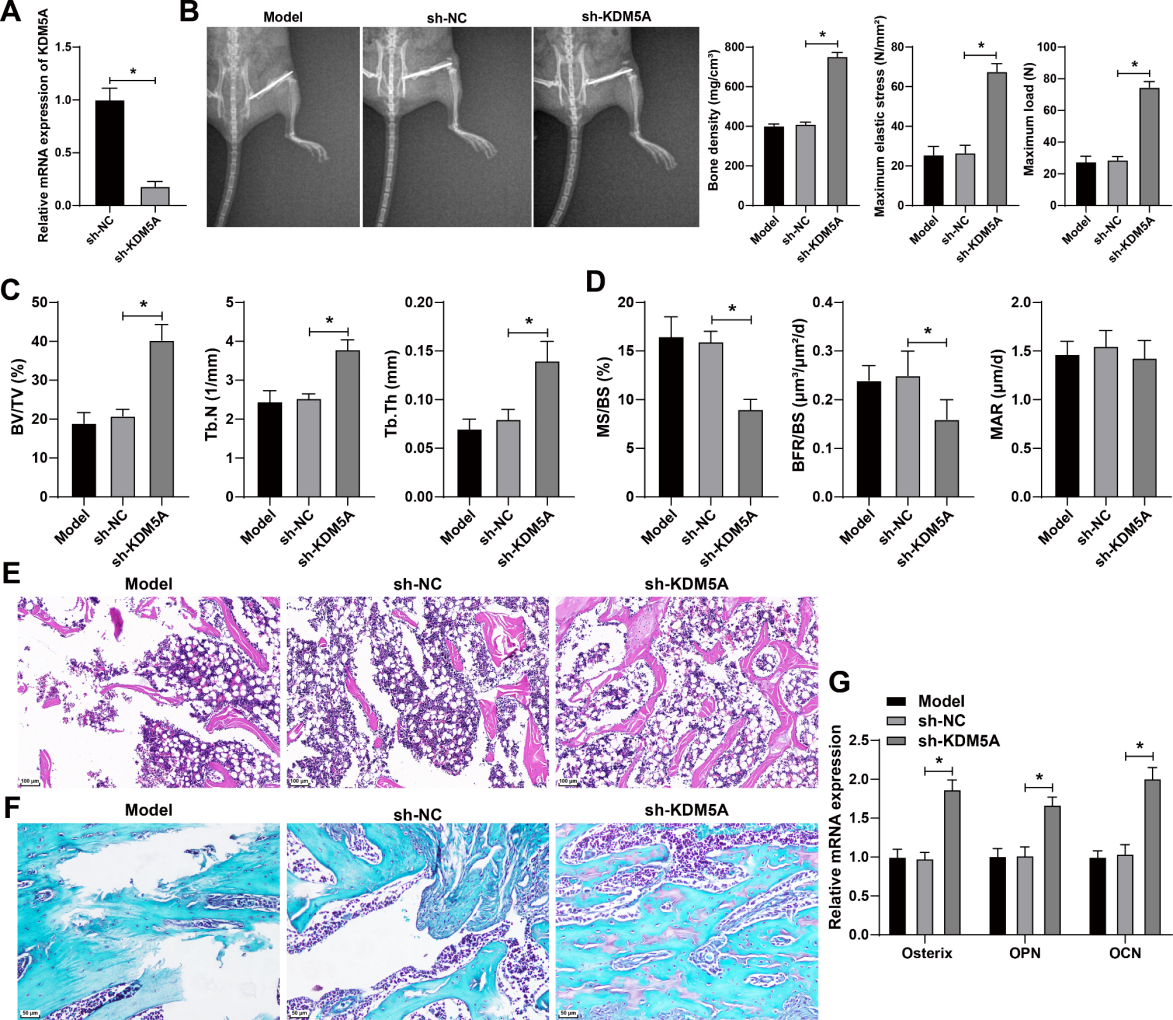
Interference of KDM5A facilitated fracture healing in osteoporotic mice. The lentiviral interference vector of KDM5A was used to interfere with KDM5A expression in model mice. **A**: The KDM5A interference efficiency was detected by qRT-PCR. **B**: The fracture was observed, and the bone mineral density, maximum elastic stress and maximum load were measured by dual-energy X-ray absorptiometry. **C**: The relative trabecular bone volume (BV/TV), bone trabecular thickness (Tb.Th), and trabecular number (Tb.N) were measured by Micro-CT. **D**: The dynamic osteogenic parameters in histomorphometry were examined. **E**: The histopathological changes of femoral tissues after modeling were observed by H&E staining. **F**: The changes of bone microstructure were observed by safranin fast green staining. **G**: The expressions of osteogenic factors including osterix, osteopontin (OPN), and osteocalcin (OCN) in tissues were detected by qRT-PCR. *P < 0.05; **P < 0.01. The measurement data were expressed by Mean ± SD, and one-way ANOVA was used for comparisons among multiple groups, followed by Tukey’s post hoc test. *N* = 8

To explore the specific effect of KDM5A on osteoporotic fracture healing, we used lentiviral interference vector of KDM5A to interfere with KDM5A expression in model mice. qRT-PCR confirmed that compared with the sh-NC group, the sh-KDM5A-1 group had the lowest KDM5A expression, so it was selected for the subsequent experiments (all *p* < 0.05) (Fig. 2A). After interference of KDM5A expression, the fracture line of mice was obviously blurred, and the BMD, maximum elastic stress, and maximum load were notably increased (all *p* < 0.05) (Fig. 2B). Interference of KDM5A enhanced BV/TV, Tb.N, and Tb.Th (all p 0.05) (Fig. 2C), and notably reduced MS/BS and BFR/BS in bone tissues (all *p* < 0.05), while there was still no statistical difference in MAR (Fig. 2D). After interference of KDM5A expression, the trabecular bone became thicker and the space between trabecular bone became smaller, with increasingly neat arrangement (Fig. 2E). The changes of bone microstructure were observed using safranin O/fast green staining. The results showed that interference of KDM5A resulted in obvious cartilage formation, cartilage osteogenesis, and trabecular bone formation (Fig. 2F). The expressions of osterix (Osx), osteopontin (OPN), and osteocalcin (OCN) were detected using qRT-PCR. The expressions of Osx, OPN, and OCN in the sh-KDM5A group were notably higher than those in the sh-NC group (Fig. 2G). Taken together, interference of KDM5A facilitated osteogenic differentiation and then osteoporotic fracture healing.

#### Interference of KDM5A accelerated proliferation, differentiation, and mineralization of osteoblasts in vitro

**Fig. 3:**
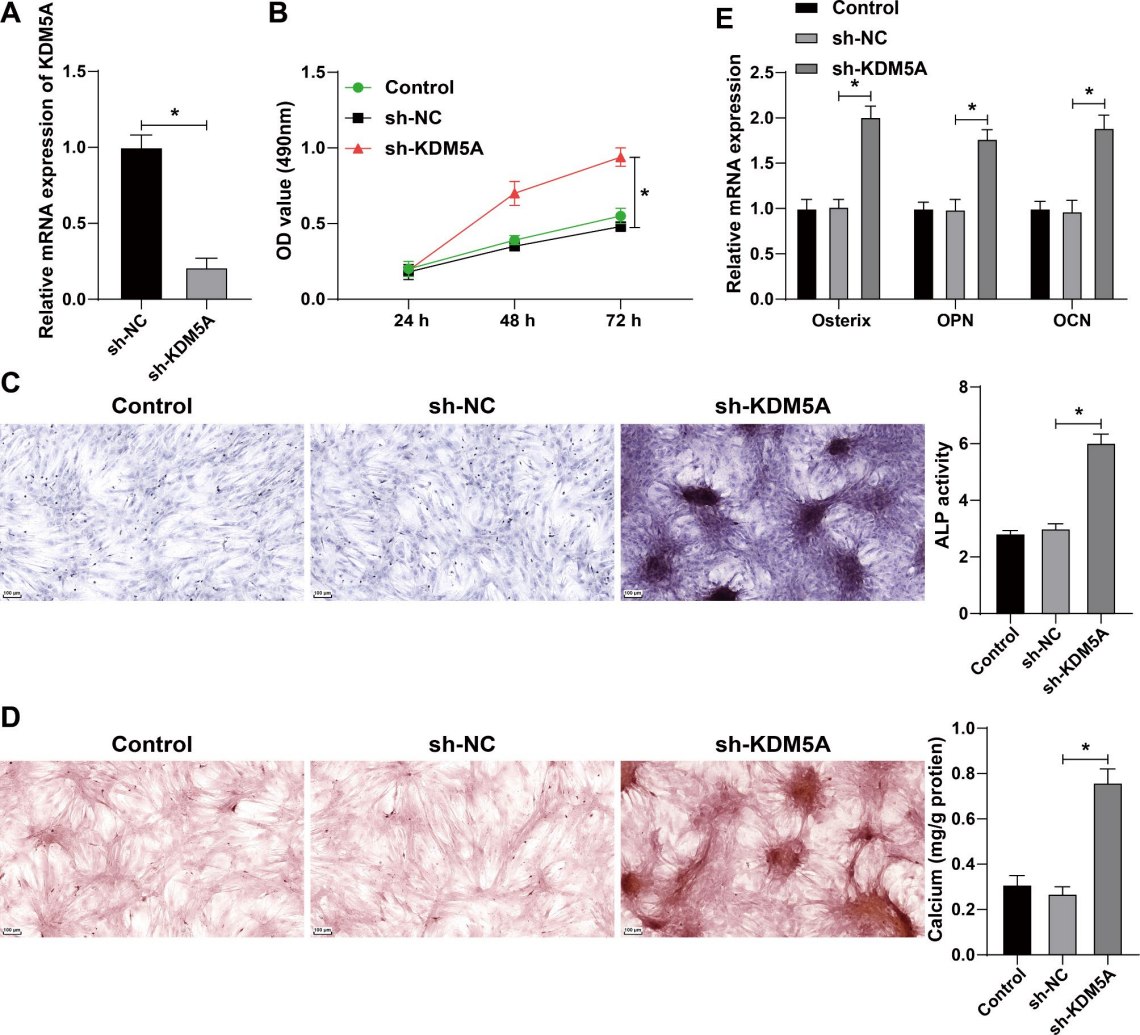
Interference of KDM5A accelerated proliferation, differentiation, and mineralization of osteoblasts in vitro. MC3T3-E1 cells were cultured in osteogenic medium and infected with lentivirus sh-KDM5A, with sh-NC as the control. **A**: The KDM5A interference efficiency was detected by qRT-PCR. **B**: The cell proliferation ability was evaluated by CCK-8 assay. **C**: The ALP ability of cells was measured by ALP staining. **D**: The number of calcified nodules in cells was detected by Alizarin red staining. **E**: The expressions of osteogenic factors including osterix, osteopontin (OPN), and osteocalcin (OCN) in cells were detected by qRT-PCR. *P < 0.05; **P < 0.01. The measurement data were expressed by Mean ± SD, and the comparison of each time point was performed by repeated measure analysis of variance; one-way ANOVA was used for comparisons among multiple groups, followed by Tukey’s post hoc test. The experiments were repeated three times independently

Then we explored the effect of KDM5A on osteogenesis in vitro. KDM5A expression in osteoblast MC3T3-E1 was successfully interfered (Fig. 3A). CCK-8 assay showed that compared with the sh-NC group, the cell proliferation ability of the KDM5A group was significantly enhanced (all *p* < 0.05) (Fig. 3B). ALP staining showed that compared with the sh-NC group, the sh-KDM5A group showed significantly enhanced ALP activity (all *p* < 0.05) (Fig. 3C). Alizarin red staining exhibited that compared with the sh-NC group, the sh-KDM5A group had the increased number of calcified nodules (all *p* < 0.05) (Fig. 3D). qRT-PCR results demonstrated that compared with the sh-NC group, the sh-KDM5A group showed notably increased expressions of Osx, OPN, and OCN (Fig. 3E). Briefly, interference of KDM5A expression accelerated proliferation, differentiation, and mineralization of osteoblasts in vitro.

#### KDM5A downregulated miR-495 expression by promoting the H3K4me3 methylation of miR-495 promoter

**Fig. 4:**
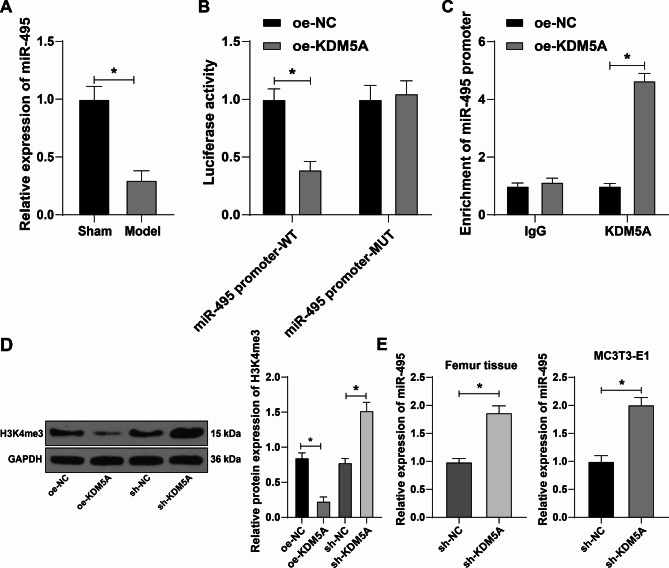
KDM5A downregulated miR-495 expression by promoting the H3K4me3 methylation of miR-495 promoter. **A**: miR-495 expression in mouse femoral tissues was detected by qRT-PCR. **B**: The binding between KDM5A and miR-495 promoter was measured by dual-luciferase assay. **C**: The enrichment of KDM5A in the miR-495 promoter after overexpression of KDM5A was detected by ChIP. **D**: The H3K4me3 level in antibody/transcription factor complex adsorbed by KDM5A antibody in ChIP experiment was detected by Western blotting. **E**: miR-495 expression in mouse femoral tissues and cells after interference of KDM5A was detected by qRT-PCR. *P < 0.05. The measurement data were expressed by Mean ± SD, and one-way ANOVA was used for comparisons among multiple groups, followed by Tukey’s post hoc test. The experiments were repeated three times independently

We used qRT-PCR to detect miR-495 expression in mouse femoral tissues. qRT-PCR showed that compared with the sham-operated mice, the model mice had significantly reduced miR-495 expression (all *p* < 0.05) (Fig. 4A), indicating that miR-495 was related to osteoporosis. Dual-luciferase assay verified that KDM5A could bind to miR-495 promoter (Fig. 4B). ChIP assay showed that miR-495 promoter enrichment was notably increased after overexpression of KDM5A (Fig. 4C). WB was used to detect the level of H3K4me3 in the complex adsorbed by KDM5A antibody in ChIP assay. It was found that H3K4me3 level was significantly declined after overexpression of KDM5A, while the H3K4me3 level was elevated after interference of KDM5A (Fig. 4D). Furthermore, miR-495 expression in femur tissues of osteoporosis mice and MC3T3-E1 cells was increased after interference of KDM5A (Fig. 4E). In brief, KDM5A downregulated miR-495 expression by promoting the H3K4me3 methylation of miR-495 promoter.

#### Inhibition of miR-495 reversed the effect of KDM5A silencing on osteoblast proliferation, differentiation, and mineralization

**Fig. 5:**
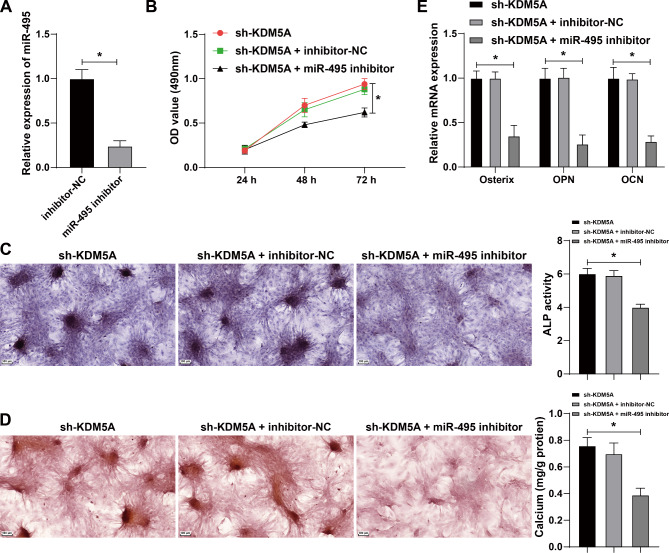
KDM5A significantly affected the biological functions of osteoblasts by regulating miR-495. MC3T3-E1 cells were cultured in osteogenic medium and transfected with miR-495 inhibitor, with inhibitor NC as the control, and then subjected to a combined experiment with sh-KDM5A. **A**: The miR-495 interference efficiency was detected by qRT-PCR. **B**: The cell proliferation ability was evaluated by CCK-8 assay. **C**: The ALP ability of cells was measured by ALP staining. **D**: The number of calcified nodules in cells was detected by Alizarin red staining. **E**: The expressions of osteogenic factors including osterix, osteopontin (OPN), and osteocalcin (OCN) in cells were detected by qRT-PCR. *P < 0.05; **P < 0.01. The measurement data were expressed by Mean ± SD, and the comparison of each time point was performed by repeated measure analysis of variance; one-way ANOVA was used for comparisons among multiple groups, followed by Tukey’s post hoc test. The experiments were repeated three times independently

To further determine whether KDM5A affected osteogenesis by regulating miR-495, we set up rescue experiments on MC3T3-E1 cells. The cells were assigned into sh-KDM5A group, sh-KDM5A + inhibitor-NC group, and sh-KDM5A + miR-495 inhibitor group. qRT-PCR confirmed the transfection efficiency of miR-495 inhibitor (Fig. 5A). CCK-8 assay exhibited that compared with the sh-KDM5A group, the sh-KDM5A + miR-495 inhibitor group had notably reduced cell proliferation ability (all *p* < 0.05) (Fig. 5B). ALP staining showed that compared with the sh-KDM5A group, the sh-KDM5A + miR-495 inhibitor group had declined ALP activity (all p < < 0.05) (Fig. 5C). Alizarin red staining exhibited that compared with the sh-KDM5A group, the sh-KDM5A + miR-495 inhibitor had the reduced number of calcified nodules (all *p* < 0.05) (Fig. 5D). qRT-PCR demonstrated that compared with the sh-KDM5A group, the sh-KDM5A + miR-495 inhibitor group showed notably decreased expressions of Osx, OPN, and OCN (Fig. 5E). Briefly, inhibition of miR-495 reversed the promoting effect of KDM5A silencing on osteoblast proliferation, differentiation, and mineralization.

### miR-495 facilitated osteoblast proliferation, differentiation, and mineralization by targeting SKP2

**Fig. 6:**
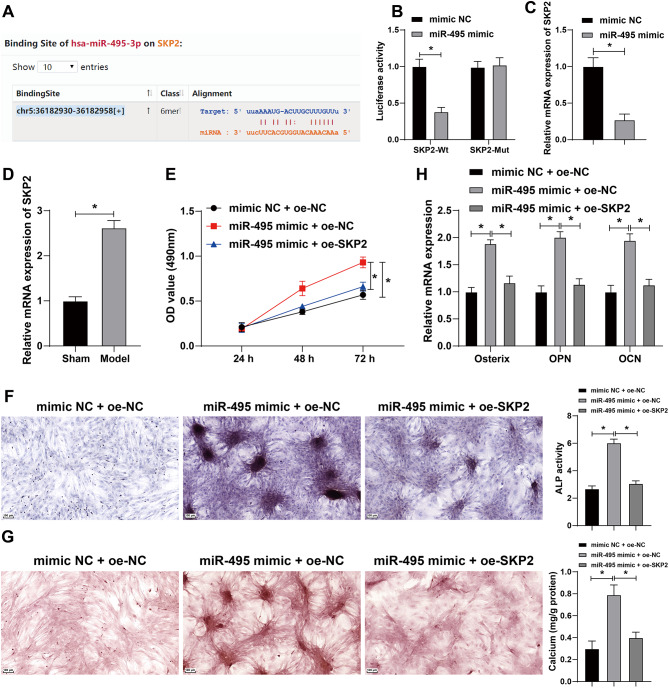
miR-495 facilitated osteoblast proliferation, differentiation, and mineralization by targeting SKP2. Cells were cultured in osteogenic medium and transfected with miR-495 mimic, with mimic NC as the control. Then, the cells were infected with lentivirus oe-SKP2, with oe-NC as the control. **A**: The binding site between miR-495 and SKP2 was predicted through bioinformatics website. **B**: The binding relationship between miR-495 and SKP2 was verified by dual-luciferase assay. **C**: SKP2 expression in cells after overexpression of miR-495 was detected by qRT-PCR. **D**: SKP2 expression in femoral tissues was detected by qRT-PCR (*N* = 8). After simultaneous overexpression of miR-495 and SKP2, **E**: The cell proliferation ability was evaluated by CCK-8 assay. F: The ALP ability of cells was measured by ALP staining. **G**: The number of calcified nodules in cells was detected by Alizarin red staining. **H**: The expressions of osteogenic factors including osterix, osteopontin (OPN), and osteocalcin (OCN) in cells were detected by qRT-PCR. *P < 0.05. The measurement data were expressed by Mean ± SD, and the comparison of each time point was performed by repeated measure analysis of variance; one-way ANOVA was used for comparisons among multiple groups, followed by Tukey’s post hoc test. The experiments were repeated three times independently

Bioinformatics website (http://starbase.sysu.edu.cn) predicted that there was a binding site between miR-495 and SKP2 (Fig. 6A). The dual-luciferase assay confirmed that miR-495 could bind to SKP2 (all *p* < 0.05) (Fig. 6B). qRT-PCR revealed that overexpression of miR-495 significantly reduced SKP2 expression in osteoblasts (all *p* < 0.05) (Fig. 6C), and SKP2 expression in femur tissues of osteoporosis mice was significantly elevated (all *p* < 0.05) (Fig. 6D). Therefore, we speculated that miR-495 affected osteoporosis by targeting SKP2 expression.

To further verify the above speculation, we assigned MC3T3-E1 cells into the following groups: mimic NC + oe-NC group, miR-495 mimic + oe-NC group, and miR-495 mimic + oe-SKP2 group. Compared with the mimic NC + oe-NC group, the miR-495 mimic + oe-NC group had enhanced cell proliferation ability; compared with the miR-495 mimic + oe-NC group, the miR-495 mimic + oe-SKP2 group had reduced cell proliferation ability (all *p* < 0.05) (Fig. 6E). Compared with the mimic NC + oe-NC group, the miR-495 mimic + oe-NC group had enhanced ALP activity; compared with the miR-495 mimic + oe-NC group, the miR-495 mimic + oe-SKP2 group had reduced ALP activity (all *p* < 0.05) (Fig. 6F). Compared with the mimic NC + oe-NC group, the miR-495 mimic + oe-NC group had the increased number of calcified nodules; compared with the miR-495 mimic + oe-NC group, the miR-495 mimic + oe-SKP2 group had the decreased number of calcified nodules (all *p* < 0.05) (Fig. 6G). Compared with the mimic NC + oe-NC group, the miR-495 mimic + oe-NC group had elevated expressions of Osx, OPN, and OCN; compared with the miR-495 mimic + oe-NC group, the miR-495 mimic + oe-SKP2 group had declined expressions of Osx, OPN, and OCN (all *p* < 0.05) (Fig. 6H). Taken together, miR-495 facilitated osteoblast proliferation, differentiation, and mineralization by targeting SKP2.

### SKP2 suppressed Runx2 expression through ubiquitination degradation

**Fig. 7:**
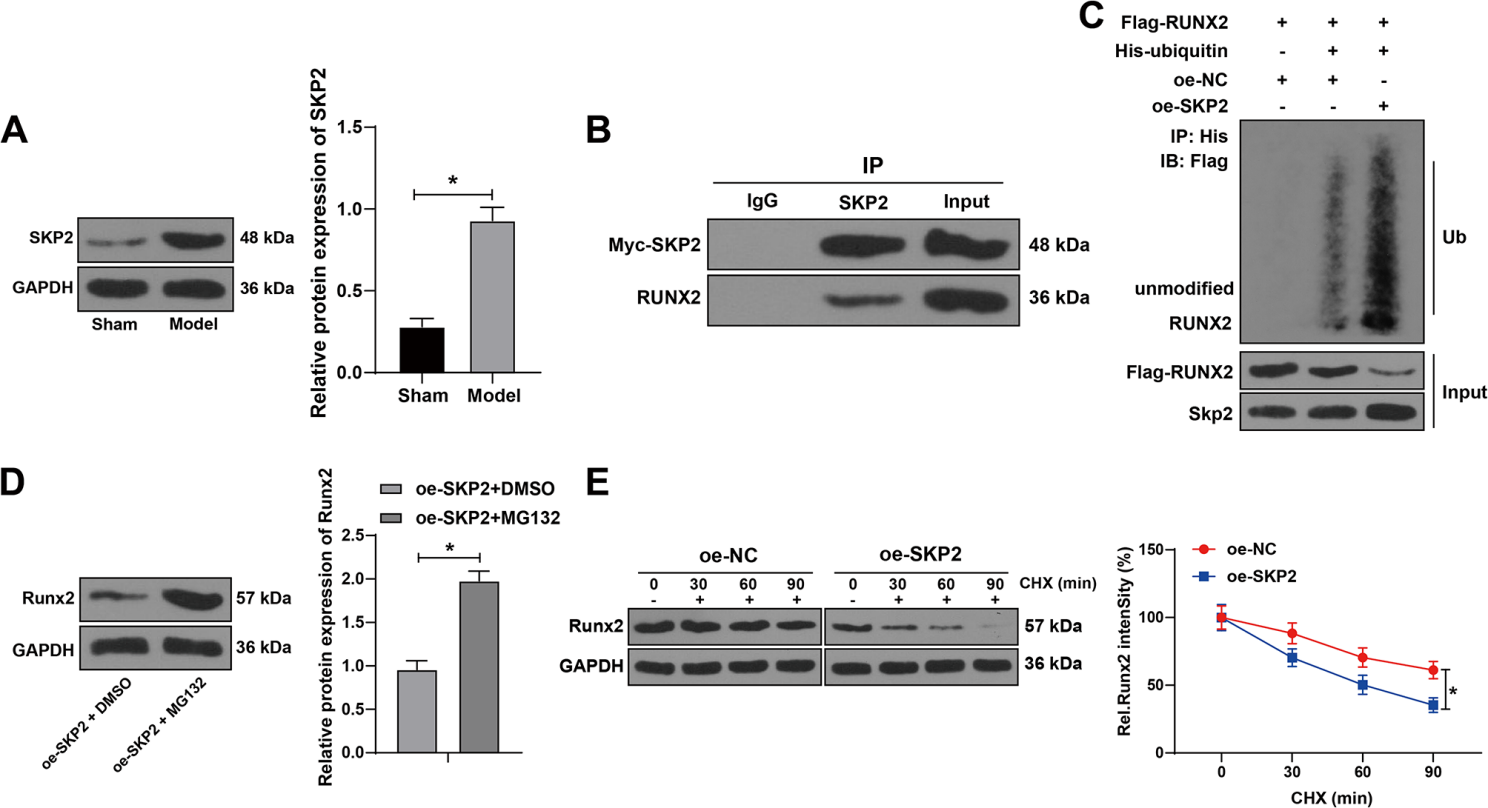
SKP2 suppressed Runx2 expression through ubiquitination degradation. **A**: The interaction between SKP2 and Runx2 was detected by IP assay. **B**: The ubiquitination level of Runx2 was detected by ubiquitin pull down. **C**: The stability of Runx2 protein was detected by cycloheximide chase assay. **D**: The cells overexpressing SKP2 were treated with MG132, a ubiquitination protease inhibitor, and the Runx2 protein expression was detected by Western blotting. E: Runx2 expression in femoral tissues was detected by Western blotting (*N* = 8). *P < 0.05. The measurement data were expressed by Mean ± SD, and the comparison of each time point was performed by repeated measure analysis of variance; one-way ANOVA was used for comparisons among multiple groups, followed by Tukey’s post hoc test. The experiments were repeated three times independently

To explore the downstream mechanism of SKP2 in osteogenic differentiation, we used WB to detect the protein level of Runx2 in femur tissues of mice. The protein level of Runx2 in model mice was notably lower than that in sham-operated mice (Fig. 7A). In IP assay, Runx2 protein could be found in the pull-down complex of antibody SKP2, indicating the interaction between SKP2 and Runx2 (Fig. 7B). Ubiquitin pull-down assay tested the ubiquitination level of Runx2, and the results revealed that the ubiquitination level of Runx2 was increased notably and the protein level of Runx2 was decreased after overexpression of SKP2 (Fig. 7C). After the addition of ubiquitin protease inhibitor MG132 to SKP2 overexpressing cells, the protein level of Runx2 was increased (Fig. 7D). Cycloheximide chase assay demonstrated that after combined treatment of oe-NC or oe-SKP2 into osteoblasts and protein synthesis inhibitor CHX, the stability of Runx2 protein was decreased after SKP2 overexpression (Fig. 7E). These results suggested that SKP2 suppressed Runx2 expression through ubiquitination degradation.

### Inhibition of Runx2 reversed the promoting effect of SKP2 silencing on osteogenic differentiation

**Fig. 8:**
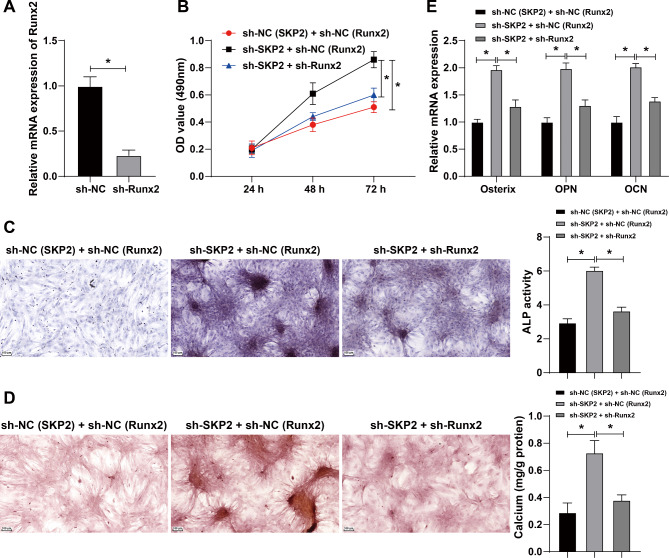
SKP2 affected osteoblast differentiation by regulating Runx2. MC3T3-E1 cells were cultured in osteogenic medium and infected with lentivirus sh-SKP2, with sh-NC (SKP2) as the control. Then, cells were infected with lentivirus sh-Runx2, with sh-NC (Runx2) as the control. **A**: The Runx2 interference efficiency was detected by qRT-PCR. After simultaneous interference of SKP2 and Runx2 expression in MC3T3-E1 cells, **B**: The cell proliferation ability was evaluated by CCK-8 assay. **C**: The ALP ability of cells was measured by ALP staining. **D**: The number of calcified nodules in cells was detected by Alizarin red staining. **E**: The expressions of osteogenic factors including osterix, osteopontin (OPN), and osteocalcin (OCN) in cells were detected by qRT-PCR. *P < 0.05; **P < 0.01. The measurement data were expressed by Mean ± SD, and the comparison of each time point was performed by repeated measure analysis of variance; one-way ANOVA was used for comparisons among multiple groups, followed by Tukey’s post hoc test. The experiments were repeated three times independently

To further verify whether SKP2 affected osteogenesis by regulating Runx2, we assigned MC3T3-E1 cells into sh-NC (SKP2) + sh-NC (Runx2) group, sh-SKP2 + sh-NC (Runx2) group, and sh-SKP2 + sh-Runx2 group. qRT-PCR confirmed the interference efficiency of Runx2 (Fig. 8A).

Compared with the sh-NC (SKP2) + sh-NC (Runx2) group, the sh-SKP2 + sh-NC (Runx2) group had enhanced cell proliferation ability; compared with the sh-SKP2 + sh-NC (Runx2) group, the sh-SKP2 + sh-Runx2 group had reduced cell proliferation ability (all *p* < 0.05) (Fig. 8B). Compared with the sh-NC (SKP2) + sh-NC (Runx2) group, the sh-SKP2 + sh-NC (Runx2) group had enhanced ALP activity; compared with the sh-SKP2 + sh-NC (Runx2) group, the sh-SKP2 + sh-Runx2 group had reduced ALP activity (all *p* < 0.05) (Fig. 8C). Compared with the sh-NC (SKP2) + sh-NC (Runx2) group, the sh-SKP2 + sh-NC (Runx2) group had the increased number of calcified nodules; compared with the sh-SKP2 + sh-NC (Runx2) group, the sh-SKP2 + sh-Runx2 group had the decreased number of calcified nodules (all *p* < 0.05) (Fig. 8D). Compared with the sh-NC (SKP2) + sh-NC (Runx2), the sh-SKP2 + sh-NC (Runx2) group had elevated expressions of Osx, OPN, and OCN; compared with the sh-SKP2 + sh-NC (Runx2) group, the sh-SKP2 + sh-Runx2 group had declined expressions of Osx, OPN, and OCN (all *p* < 0.05) (Fig. 8E). Taken together, inhibition of Runx2 reversed the promoting effect of SKP2 silencing on osteogenic differentiation.

## Discussion

**Fig. 9:**
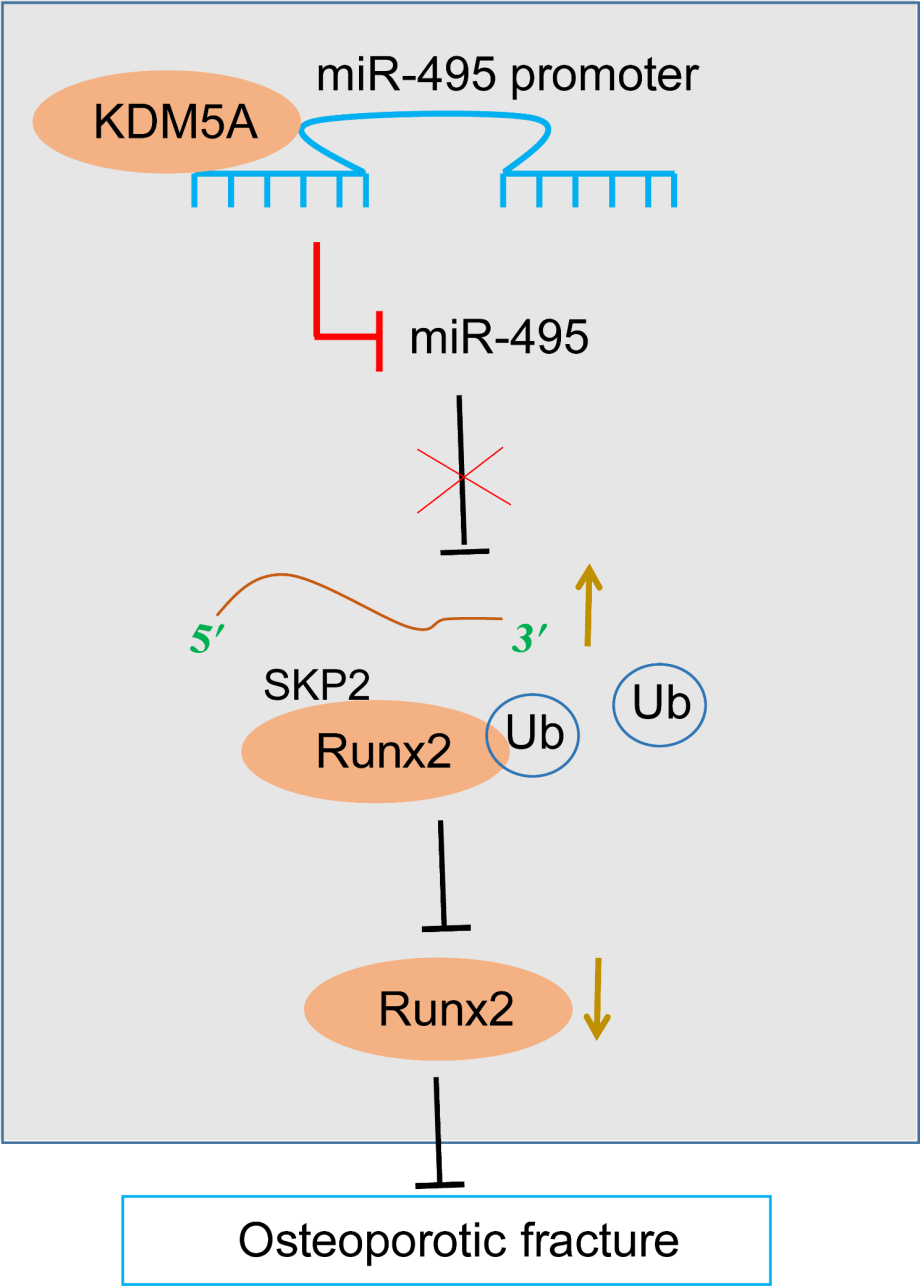
KDM5A downregulates miR-495 expression by promoting H3K4 trimethylation of miR-495 promoter to attenuate the inhibitory effect of miR-495 on SKP2, thereby upregulating the expression of SKP2 and promoting the ubiquitination degradation of Runx2 protein by SKP2 to inhibit osteoblast differentiation and delay osteoporotic fracture healing

The increased risk of fracture caused by osteoporosis constitutes one of the most salient disability factors in postmenopausal women (Jackson and Mysiw 2014). Histone demethylase can regulate bone mass (Sun et al. 2018) and osteogenic differentiation MSCs during osteoporosis (Ye et al. 2012; Wang et al. 2016b). This study demonstrated that histone demethylase KDM5A retarded osteoporotic fracture healing through epigenetic regulation of the miR-495/SKP2/Runx2 axis (Fig. 9).

KDM5A-mediated H3K4me3 demethylation is reported to participate in the pathogenesis of osteoporosis (Zhang et al. 2020). Wang et al. have demonstrated the role of KDM5A in suppressing bone formation in osteoporotic mice, and KDM5A inhibitor treatment partly rescues bone loss during osteoporosis (Wang et al. 2016b). Nevertheless, the exact role of KDM5A in osteoporotic fracture healing has not been fully identified. In this study, the murine model of osteoporotic fracture was established, and KDM5A was found to be highly expressed in the femur tissues of mice with osteoporotic fracture. To explore the effect of KDM5A on osteoporotic fracture healing, we used lentivirus interference vector of KDM5A to treat the model mice. After interference of KDM5A expression, the fracture line of mice was obviously blurred, and the BMD, maximum elastic stress, and maximum load were notably increased. Interference of KDM5A enhanced BV/TV, Tb.N, and Tb.Th, and notably reduced MS/BS and BFR/BS in bone tissues. After interference of KDM5A expression, the trabecular bone became thicker and the space between trabecular bone became smaller, with increasingly neat arrangement. Interference of KDM5A resulted in obvious cartilage formation, cartilage osteogenesis, and trabecular bone formation, as well as increased osteogenic factors. Moreover, we explored the effect of KDM5A on osteogenesis in vitro. Interference of KDM5A in MC3T3-E1 cells elevated ALP activity, increased calcified nodule, and enhanced the expressions of osteogenic factors, suggesting that interference of KDM5A in vitro facilitated the proliferation, differentiation, and mineralization of osteoblasts. Consistently, KDM5A represses odontogenic differentiation of human dental pulp cells by removing H3K4me3 from the specific gene promoter (Li et al. 2020). Knockdown of KDM5A elevates H3K4me3 level and enhances osteogenic differentiation of BMSCs (Zhang et al. 2020). KDM5A depletion contributes to enhancing ALP activity and mineral deposition formation (Li et al. 2020). In brief, these findings revealed that interference of KDM5A facilitated osteogenic differentiation and osteoporotic fracture healing.

Subsequently, we explored the downstream mechanism of KDM5A regulating osteoporosis in MC3T3-E1 cells. A previous literature has pointed out that KDM5A can bind to miR-495 promoter, resulting in the inhibition of its transcription and expression (Du et al. 2020). miR-495 is involved in osteoblastic differentiation of MSCs (Xu et al. 2017), and miR-495 can promote bone differentiation by upregulating osteoprotegerin in human fibroblast-like synovial cells (Du et al. 2019). The role of miR-495 in osteoporotic fracture has not been reported before. To our knowledge, we were the first to report the downregulation of miR-495 in osteoporosis mice. Dual-luciferase assay verified that KDM5A could bind to miR-495 promoter. miR-495 promoter enrichment was notably increased after overexpression of KDM5A. miR-495 expression in femur tissues of osteoporosis mice and MC3T3-E1 cells was increased after interference of KDM5A. H3k4me3 level was significantly declined after overexpression of KDM5A, while h3k4me3 level was elevated after interference of KDM5A. These results indicated that KDM5A downregulated miR-495 expression in osteoporosis by promoting the H3K4me3 methylation of miR-495 promoter. Inhibition of miR-495 reversed the effect of KDM5A on osteoblast proliferation, differentiation, and mineralization. Zhu et al. have also revealed that miR-495 can facilitate proliferation and differentiation of osteoblasts in mice with tibial fracture by activating the p38 MAPK pathway (Zhu et al. 2019). Bioinformatics website predicted that there was a binding site between miR-495 and SKP2. SKP2, as a type of E3 ubiquitin ligases, can induce ubiquitination and subsequent proteasome-dependent degradation of its target substrates and then confer a driving force for variant cellular functions (Asmamaw et al. 2020). SKP2 targets Runx2 by enhancing its polyubiquitination and proteasome-dependent degradation, and downregulation of Runx2 mediated by SKP2 leads to impaired osteoblast differentiation (Thacker et al. 2016). Runx2 is recognized as a critical transcription factor of skeletal development during embryogenesis (Yin et al. 2018), which is essential for bone formation and osteoblast differentiation (Kim et al. 2020). Emerging evidences have unveiled that Runx2 is downregulated in osteoporosis, participating in osteoblast differentiation and osteoporosis pathogenesis (Huang et al. 2020; Cheng et al. 2019; Cai et al. 2020). Our results demonstrated that miR-495 facilitated osteoblast proliferation, differentiation, and mineralization by targeting SKP2. There was an interaction between SKP2 and Runx2. The ubiquitination level of Runx2 was increased notably and the protein level of Runx2 was decreased after overexpression of SKP2. After the addition of ubiquitin protease inhibitor MG132 to SKP2 overexpressing cells, the protein level of Runx2 was increased. Briefly, SKP2 suppressed Runx2 expression in osteoporosis through ubiquitination degradation. We performed functional rescue experiments to further verify whether SKP2 affected osteogenesis by regulating Runx. The results confirmed that inhibition of Runx2 reversed the promotion of SKP2 silencing on osteogenic differentiation. Downregulated RUNX2 suppresses osteoblast differentiation and bone formation in osteoporosis (Li et al. 2019a). Deletion of Runx2 results in the reduction of bone mass, which is related to impaired bone formation and excessive bone marrow adiposity (Tosa et al. 2019).

To sum up, KDM5A attenuated the inhibition of miR-495 on SKP2 and promoted the ubiquitination degradation of Runx2 protein by SKP2, thus repressing osteoblast differentiation and retarding osteoporotic fracture healing. This fundamental information can provide us with a deeper understanding of the pathogenesis and treatment strategies of osteoporosis. This study also has some limitations. Due to limitations in laboratory funding and conditions, we are currently unable to conduct relevant microarray analysis to detect differentially expressed genes and dissect the mechanistic signaling pathways. Moreover, rescue experiments failed to be conducted in vivo. In the future study, we will focus on verifying the mechanism of KDM5A in vivo and exploring the possible signaling pathway involved in it.

## Data Availability

The datasets used and/or analyzed during the current study are available from the corresponding author upon reasonable request.

## Abbreviations

KDM5A: Lysine-specific demethylase 5 A
SKP2: S-phase kinase-associated protein 2
Runx2: Runt-related transcription factor 2
BMSC: Bone mesenchymal stem cell
miRNAs: MicroRNAs
Micro-CT: Micro computed tomography
BMD: Bone mineral density
NC: Negative control
H&E: Hematoxylin and eosin
BV/TV: Relative trabecular bone volume
Tb.Th: Bone trabecular thickness
Tb.N: Trabecular number
MAR: Mineral apposition rate
MS/BS: Ratio of mineralized surface to bone surface
BFR/BS: Bone formation rate
CCK-8: Cell counting kit-8
α-MEM: α-modified Eagle’s medium
FBS: Fetal bovine serum
LPS: Lipopolysaccharide
ALP: Alkaline phosphatase
BCA: Bicinchoninic acid
qRT-PCR: Quantitative real-time polymerase chain reaction
PBS: Phosphate buffer saline
DMEM: Dulbecco’s modified Eagle’s medium
ChIP: Chromatin immunoprecipitation
RIPA: Radio-immunoprecipitation assay
IP: Immunoprecipitation
PMSF: Phenylmethylsulfonyl fluoride
GAPDH: Glyceraldehyde-3-phosphate dehydrogenase
ANOVA: Analysis of variance
